# The Roles of Kidney-Resident ILC2 in Renal Inflammation and Fibrosis

**DOI:** 10.3389/fimmu.2021.688647

**Published:** 2021-07-26

**Authors:** Ryuichi Nagashima, Masayuki Iyoda

**Affiliations:** ^1^ Department of Microbiology and Immunology, Showa University School of Medicine, Tokyo, Japan; ^2^ Division of Nephrology, Department of Medicine, Showa University School of Medicine, Tokyo, Japan

**Keywords:** ILC2, renal fibrosis, CKD - chronic kidney disease, ILCreg, IL-33

## Abstract

Innate lymphoid cells (ILCs) are a recently discovered lymphocyte population with high cytokine productive capacity. Type-2 ILCs (ILC2s) are the most studied, and they exert a rapid type-2 immune response to eliminate helminth infections. Massive and sustainable ILC2 activation induces allergic tissue inflammation, so it is important to maintain correct ILC2 activity for immune homeostasis. The ILC2-activating cytokine IL-33 is released from epithelial cells upon tissue damage, and it is upregulated in various kidney disease mouse models and in kidney disease patients. Various kidney diseases eventually lead to renal fibrosis, which is a common pathway leading to end-stage renal disease and is a chronic kidney disease symptom. The progression of renal fibrosis is affected by the innate immune system, including renal-resident ILC2s; however, the roles of ILC2s in renal fibrosis are not well understood. In this review, we summarize renal ILC2 function and characterization in various kidney diseases and highlight the known and potential contributions of ILC2s to kidney fibrosis.

## Introduction

Kidney fibrosis is a critical condition leading to kidney dysfunction and is a common characteristic of chronic kidney diseases (CKDs), which are increasing around the world (1). The major clinical issue in the progression of renal fibrosis is the loss of kidney function, which requires dialysis or kidney transplantation in end-stage renal disease (ESRD) (2). Kidney injuries, such as acute kidney injury (AKI) or glomerulonephritis, contribute to the progression of kidney fibrosis and CKD pathology. Environmental factors including metabolic syndrome, diabetes, and hypertension are also risk factors for renal disease onset and progression. Recently, an AKI-to-CKD continuum has been recognized as a clinical issue that contributes to fibrosis (3). Therefore, the establishment of therapies for renal fibrosis will improve quality of life not only for kidney disease patients but also for various tissue fibrosis patients.

Tissue fibrosis involves several causal factors, such as epithelial- and endothelial-mesenchymal transition and the immune system (4, 5). In the kidneys, fibroblasts in renal stroma transform to myofibroblasts by profibrotic factors such as TGF-β, PDGF, FGF2, and CTGF, and express the myofibroblast-unique markers α-SMA and fibronectin (6–8). These profibrotic factors are considered to derive from inflammation-induced infiltrating macrophages and migrating Tregs that repair tissue damage (9, 10). TGF-β derived from kidney-infiltrated M2 macrophages and Tregs enhances renal fibrosis (10–12). Furthermore, it has been reported that newly identified innate lymphoid cells, ILCs, are associated with tissue fibrosis, including in the lung, liver, and intestine (13–16). In kidneys, ILC2s have a protective function against AKI and glomerulonephritis, but it remains unclear if they are involved in kidney fibrosis.

As an ILC2-activating cytokine, IL-33 is a member of the IL-1 family and has been recognized as an “alarmin” that is ubiquitously expressed in various tissue cells (17, 18). IL-33 protein is divided into three domains, a nuclear domain, central domain, and IL-1-like cytokine domain (19, 20). IL-33 is constitutively distributed in the nuclei of epithelial cells under basal conditions by binding the histone H2A-2B dimer and chromatin-binding motif within the nuclear domain (21). Upon the initiation of inflammation, stored full-length IL-33 is released quickly from nuclei, and infiltrated inflammatory cell-derived proteases act on the cleavage site in the central domain (22). Cleaved-IL-33 has high activity and binds with ST2 (IL-33 receptor)-expressing cells, leading to the induction of MyD88-IRAK-TRAF signaling for proliferation, survival, and cytokine production (18, 23). IL-33-ST2 signaling is upregulated by various kidney injuries and diseases, leading to the activation of ILC2s in the kidney (24–27); however, it likely depends on the amount of IL-33 whether ILC2s play a protective or progressive role in renal disease. Recent studies have demonstrated that renal ILC2s have pivotal roles in various kidney diseases and tissue fibrosis and repair (28–33), so that these cells are being focused on as a new therapeutic target. Here, we highlight recent findings on renal ILC, especially ILC2s, in kidney disease leading to kidney fibrosis.

## ILC Subsets

ILCs lack antigen-specific receptors, like T-cell receptors (TCRs) and B-cell receptors (BCRs), and do not express classical immune cell lineage markers. ILCs are activated depending on cytokines in the surrounding tissue microenvironment and play pivotal roles in the protection against infection, inflammation, and in immune-homeostasis (34). ILCs are categorized into three groups depending on their function: ILC1, ILC2, and ILC3, which reflect the acquired immunity of helper T-cell subsets Th1, Th2, and Th17 respectively. T-bet-expressing ILC1 exerts type-1 immune responses for viral infection, GATA3-expressing ILC2s exert type-2 immune responses for helminth infection, and ROR*γ*t-expressing ILC3s exert type-17 immune responses for bacterial infection. However, it has been clarified that ILCs are also involved in the pathogenesis of a variety of diseases. In particular, pulmonary ILC2s have critical roles in asthma accompanied with steroid resistance (35, 36). The appropriate regulation of ILCs is therefore important for controlling various diseases and maintaining immune-homeostasis. Recently, a new subset of ILCs, regulatory ILCs (ILCreg), has been reported (37). ILCregs exert immune-suppressive functions by producing IL-10 and TGF-β, similar to Tregs. Although ILC1, 2, and 3 commonly develop from innate lymphoid cell progenitors (ILCP), ILCregs differentiate from common helper innate lymphoid progenitors (CHILP) in an Id3-dependent manner (37). As ILCregs do not express the Treg master regulator foxp3, it is unclear whether ILCregs are an independent subset like Tregs. However, ILCregs have the potential for unique phenotypes and functions in comparison with Tregs, and these will be further investigated in the future.

## ILC2’s Function and Regulation

ILC2s reside in various tissues such as lung, intestine, mesenteric fat associated lymphoid cluster (FALC), liver, skin, and kidney, and are mainly responsible for helminth elimination mediated by the type-2 immune response. Upon tissue damage by allergen and pathogen exposure, IL-33, IL-25, and TSLP, which are the strongest activating cytokines for ILC2 proliferation and cytokine production, are released from epithelial cells leading to rapid activation of ILC2s. Then, activated ILC2s secret large amounts of type-2 cytokines IL-5 and IL-13, and induce eosinophilic inflammation and mucosal hyperplasia. However, abnormal and sustained ILC2 activation elicits allergic diseases such as asthma, atopic dermatitis, and rhinitis (38–40), and thus it is clinically important to understand ILC2 regulation. In addition, ILC2s also produce amphiregulin (Areg) and IL-9, which contribute to remodeling and repair of tissue damage after inflammation. Areg produced from ILC2s promotes epithelial proliferation and differentiation for epithelial repair (41). Furthermore, IL-9-producing ILC2s help resolve inflammation in rheumatoid arthritis (42).

Although IL-33 and ST2 signaling are critical in ILC2 activation, other stimulations including common *γ* chain (*γ*c) cytokines (IL-2, -7, -9, 15) and co-stimulatory molecules (ICOS, GITR, PD-1) are required for ILC2 regulation (43–45). Numerous studies have identified positive or negative regulators of ILC2s as follows: cytokines (IL-25, TSLP, IFN-*γ*, IL-27), neuropeptides (VIP, NMU, CGRP), neurotransmitters (catecholamine, acetylcholine), lipid mediators (prostaglandins and lipoxins from the arachidonic acid pathway), hormones (androgen and estrogen), and nutrients (vitamins A and D and butylate) (46–55). Since ILC2s are distributed in various tissues, it is assumed that tissue-specific regulatory mechanisms and factors of ILC2s exist. We previously reported that the oxidative-stress responder Nrf2 activates lung-ILC2s, and their activation ameliorates lung allergic inflammation (56). Oxidative stresses are frequently generated in kidney injury and disease, and thus renal ILC2s may be regulated by the Keap1-Nrf2 pathway. Taken together, ILC2s are regulated by various factors, and have diverse roles depending on the tissue environment.

## Renal ILC2 and Diseases

**Figure 1:**
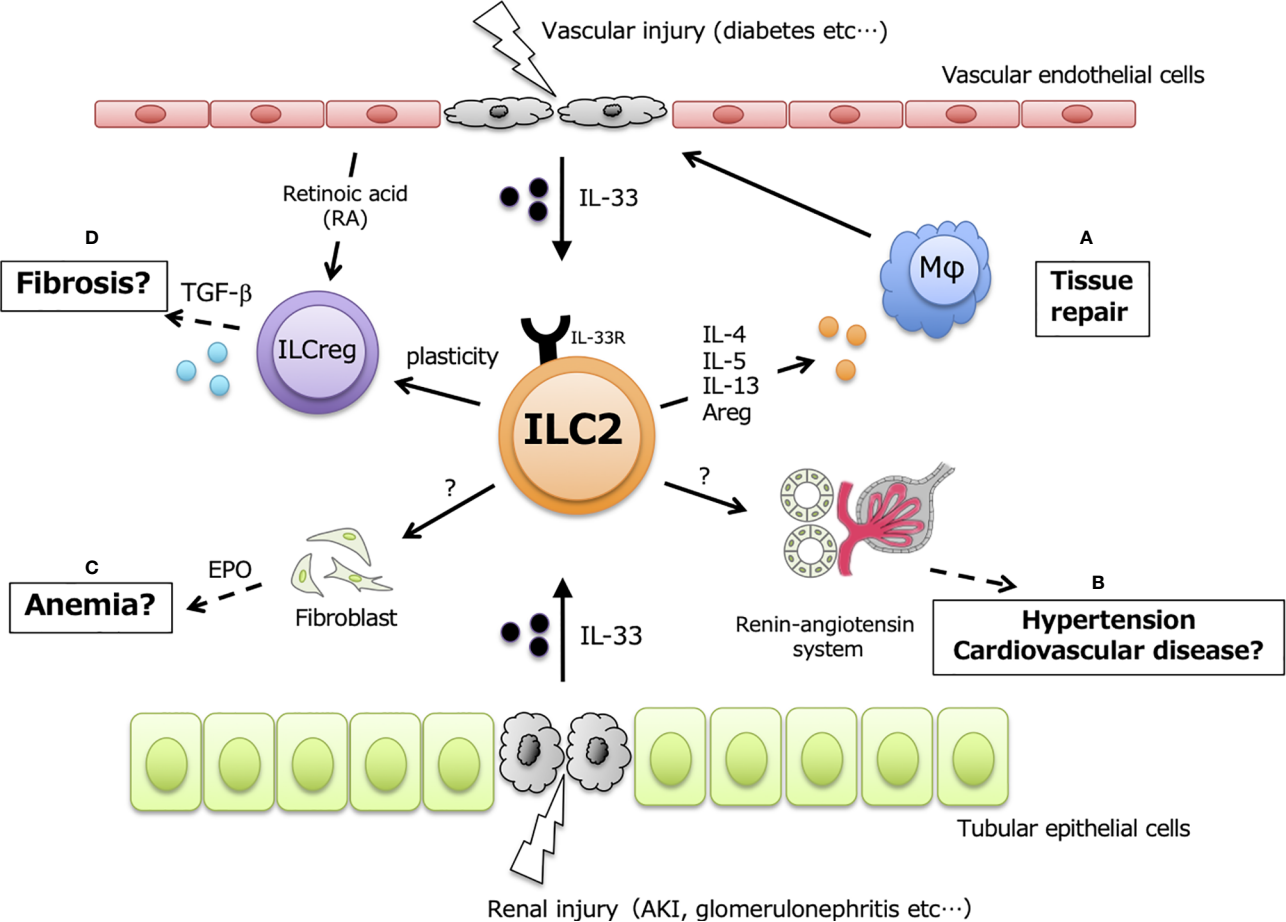
Potential functions in renal ILC2. ILC2 have protective functions in kidney, but exerts positively or negatively effects in dependent on the amount of renal IL-33. Upon kidney injury and disease, IL-33 released from vascular endothelial cells and/or tubular epithelial cells leads to activate renal-resident ILC2, and secreted type2 cytokines possibly affects in following events; **(A)** tissue repair by inducing M2 macrophages, **(B)** hypertension and cardiovascular disease through renin-angiotensin system, **(C)** renal anemia mediated by EPO, **(D)** renal fibrosis *via the* plasticity for TGF-β producing ILCreg.

ILC2s are also resident throughout murine kidneys and are especially localized in renal vasculature. GATA3-expressing ILC2s are the main ILC subset (70~80% of ILCs) in murine kidney, while T-bet-expressing ILC1s and ROR*γ*t-expressing ILC3s are less than 10% of ILCs (2 7). Renal ILC2s constitutively express IL-5 and IL-13 under steady-state conditions, and almost all of the expressed IL-5 is derived from ILC2s and not Th2 (57). Although ILC2s account for approximately 1% of total renal leukocytes, IL-33 expression is upregulated in several kidney disease models, indicating that renal ILC2s are potentially activated and exert unknown functions at both the acute and chronic phases (**Figure 1**).

AKI manifests as acute dysfunction of kidneys, inducing electrolyte imbalance, and is related to CKDs, fibrosis, and cardiovascular diseases. AKIs are caused by traumatic injury, reduced renal perfusion due to surgery, and various renal and vascular diseases (58). AKIs have been investigated using experimental AKI animal models induced by methods such as drugs, ischemia-reperfusion injury (IRI), and sepsis (59). Recently, it has been reported that ILC2s and IL-33 are associated with AKI pathogenesis. In a cisplatin-induced AKI model, Akcay et al. demonstrated that recombinant IL-33 administration exacerbates AKI, while soluble ST2, which binds IL-33 preferentially to neutralize its activity, ameliorates its pathogenesis (60). High-dose IL-33 administration induces AKI progression in a CD4^+^ T cell-dependent manner. Conversely, low-dose IL-33 has a protective effect against AKI. IRI models are commonly used to identify the mechanisms responsible for AKI pathogenesis and show that the innate immune response has a critical role. Cao et al. found that pretreatment with IL-33 ameliorates renal damage and recovers kidney function in IRI-induced mice (29). Renal ILC2s are increased in Rag1-knockout mice by the administration of IL-33, and result in reduced tubular injury score and serum creatinine irrespective of acquired immunity. However, ILC reduction using an anti-CD90 antibody in Rag1-KO mice does not rescue tubular damage. Furthermore, adaptive transfer of *ex vivo* proliferated renal ILC2s ameliorates renal injury. These results indicate that abundant ILC2s in the kidney have a renal protective effect and improve kidney functions in AKI.

CKDs have different origins such as diabetes, hypertension, and immune and toxic responses (1). Various pathologies including chronic inflammation and renal fibrosis are associated with the underlying causes of CKDs. It has been clarified that renal ILC2s have important roles in both AKI and CKD pathology. IL-33 also relieves glomerular injury by lupus nephritis (61). Moreover, the type-2 immune response induced by IL-25 and the induction of M2 macrophages can alleviate renal damage in adriamycin-induced nephropathy, which is a widely used CKD model (28). These protective effects require eosinophils recruited by IL-5 produced from ILC2s, and IL-33 fails to protect kidney function despite ILC2 abundance in eosinophil-deficient ΔdblGATA mice. However, eosinophils are considered to be pro-inflammatory cells in various diseases, so it is unclear whether eosinophil accumulation is protective against renal damage without clarifying the mechanism. In addition, ILC2s are retained in murine kidneys for up to eight weeks by IL-33 administration for four consecutive days, so there is a clinical benefit to sustained activation of ILC2s for CKD therapy. Interestingly, IL-233, a fusion cytokine of IL-2 and IL-33, contributes to kidney protection from diabetic nephropathy (30). IL-233 attenuates hyperglycemia and proteinuria in BTBR.Cg-Lep^ob/ob^ mice and has therapeutic potential for type-2 diabetic nephropathy. These findings imply that ILC2s are a potential therapeutic target in AKI and CKDs.

In humans, ILCs (lineage^-^ CD127^+^ CD161^+^ populations) account for 0.5% or fewer of total kidney lymphocytes (28). In contrast to murine kidneys, human ILC2s account for 40% or fewer of the ILCs in kidneys, and ILC3s are the main constituents. It is unclear how this difference in the ILC constitutions of human and mouse kidneys affects kidney homeostasis and disease pathogenesis. A recent study indicated that blood ILC2s are upregulated in ESRD patients (62); however, it is unclear if this elevation is related to ESRD pathogenesis. Moreover, it has also been reported that changes of blood ILC correlate with the severity of DN and LN (63, 64). Further investigation is required to determine whether human renal ILC2s are friend or foe in kidney diseases.

## ILC2 Contribute to Progression of Renal Fibrosis?

ILC2 also has been shown to affect various tissue fibrosis. ILC2 contributed collagen deposition *via* IL-25 leading to induce pulmonary fibrosis (13). IL-33 is a profibrotic cytokines, and promote the initiation and progression of lung fibrosis at ST2-dependent manner (65). Some study reported that liver-resident or cardiac tissue resident ILC2 are associated with hepatic or cardiac fibrosis, respectively (66, 67). Therefore, ILC2-IL33 axis is likely affect to promote tissue fibrosis.

Kidney fibrosis is characterized by aberrant accumulation of extracellular matrix (ECM), and then destruct robust kidney structure and function (68). Fibrosis is a part of normal response to restore tissue structure and environment. Upon kidney injury, damaged tubular and vascular epithelial cells, and infiltrated immune cells are released profibrotic factors with progression of renal damages, and then various signaling are activated leading to promote fibroblast to a-SMA positive myofibroblast transition. Persistent renal damages disrupt the balance of ECM production and degradation, and excessive ECM accumulation leads abnormal fibrosis, resulting kidney dysfunction. Progression of renal fibrosis elicit CKD exacerbation, and its pathology proceed irreversible course if kidney function less of a certain level, resulting in ESRD. In addition, renal stroma produces the erythropoietin (EPO) required for erythrocyte development, and its reduction induced by kidney fibrosis results in renal anemia. Thus, the overcome of renal fibrosis is a clinical importance in nephrology.

**Figure 2:**
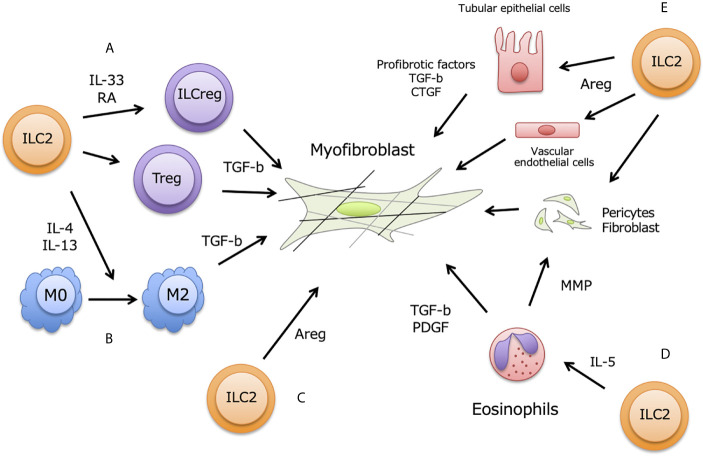
ILC2 contribution for renal fibrosis. Renal fibrosis is partially induced by pro-fibrotic factors including TGF- β from M2 macrophages, Tregs, and ILCs, and fibroblasts accelerate trans-differentiation to myofibroblasts in renal stromal region. Activation of myofibroblast occurs excessive ECM deposition, leading to impair renal functions. Renal ILC2 possibly contribute to enhance fibrosis at following aspects of myofibroblast activation; **(A)** ILC2-ILCreg plasticity, **(B)** M2 macrophages differentiation, **(C)** direct effect by Areg production, **(D)** indirect effect *via* eosinophils induced by IL-5, **(E)** Effect of Areg to produce pro-fibrotic factors on tubular epithelial cells, vascular endothelial cells, fibroblasts and pericytes.

During chronic renal injury and inflammation, damaged renal and vascular endothelial cells also release IL-33 together with aberrant ECM production, and renal-resident ILC2 are thought to be activated (**Figure 2**). The commonly used method to study renal fibrosis is unilateral ureter obstruction (UUO)-model, and IL-33 increased in serum and urine in this model (24, 69). In fact, ST2+ innate immune cells are increased in UUO-model, and ILC2 numbers also increased in murine kidney (25). Furthermore, Liang et al. showed that renal IRI-induced fibrosis is accelerated by exogenous IL-33 treatment, and soluble ST2 ameliorated fibrosis (70). High-dose administration of IL-33 promoted renal fibrosis *via* AKI, but the inhibition of IL-33 was decreased AKI-induced renal fibrosis (60). While, low-dose and short-term IL-33 administration attenuate renal damages induce by IRI (28, 29). These may imply that modest IL-33 release is induced by mild renal damages at early time point to protect renal damages, while progressive renal destruction caused excessive and long-term IL-33 release leading to exacerbate renal damages and fibrosis. Taken together, renal ILC2 and adequate IL-33 has pivotal roles in kidney fibrosis.

ILC2s have been reported to produce amphiregulin (Areg) to recover pulmonary epithelial integrity at viral infection (71, 72). In kidney, ILC2-producing Areg also exerted protective function for renal damages by IRI, and directly contribute to repair renal tubular structure (29). Knock-out of Areg in ex-vivo cultured ILC2 using by CRISPER-Cas9 system could not restore tubular injury score and serum creatinine, and renal TECs apoptosis. This renoprotective effect is partially responsible for anti-inflammatory M2 macrophages induced by activated ILC2. While, Areg has function to progress tissue fibrosis including liver, lung, and kidney (73–75). Recent studies indicated that Areg-EGFR signaling enhanced renal fibrosis in proximal renal tubules (76, 77). In addition, type2 immune responses are also profoundly associated with fibrosis. BM-derived CD11c+ cell produces Areg in response to tissue damages (78), and these producing Areg induce fibroblast activation leading to promote pulmonary fibrosis. Moreover, Areg-producing pathogenic memory Th2 cells trained eosinophils to produce large amount of osteopontin, and accelerated pulmonary fibrosis (79). In addition, Liu et al. have been reported that ILC2 was negatively correlated with eGFR level in diabetic kidney disease patient with promoting renal fibrosis (63). Although it is not well understood whether Areg produced from ILC2 contribute to progress renal fibrosis, Areg expression is required for fibrosis induced by TGF-b overproduction. Further studies would be clarified the relationship among ILC2, Areg and TGF-b leading to reveal the roles of ILC2 in renal fibrosis. Areg is detected from serum and urine from CKD and AKI patients (77), and it will be as a novel therapeutic target and biomarkers in kidney fibrosis and kidney diseases including CKD and AKI.

## A Possible New Player ILCreg in Renal Fibrosis

One cause of renal fibrosis is TGF-β signaling, which accelerates fibroblast transformation to myofibroblasts in renal stroma (80). Kidney-infiltrating macrophages and Tregs contribute to the progression of renal fibrosis *via* TGF-β production. Also, IL-4- and IL-13-producing ILC2s are associated with the progression of renal fibrosis through the induction of M2 macrophages (81). Moreover, Wang et al. reported that TGF-β signaling induces ST2 expression and contributes to the development of ILC2s from ILC2 progenitors (82). Therefore, the relationship among M2 macrophages, Tregs, and ILC2s is critical in renal fibrosis, and TGF-β plays a central role in these profibrotic networks.

The recently defined ILCreg subset resides in both human and mouse kidneys, and is a source of TGF-β in kidneys (83). Renal ILCregs suppress immune responses by secreting IL-10 and TGF-β, and they express CD25, ICOS, and transcriptional factor Id3, but not ST2 and KLRG1. *In vitro* cultured ILCregs produce large amounts of IL-10 and TGF-β and suppress the innate immune response of ILC1 and macrophages. Adaptive transfer of *ex vivo* expanded ILCregs to IRI-treated mice ameliorates kidney damage, so ILCregs have therapeutic potential for kidney disease. However, it is a concern that large amounts of TGF-β produced from ILCregs could enhance renal fibrosis, so additional studies are needed. Intriguingly, Morita et al. demonstrated that ILC2s are plastic and can develop into ILCregs in human nasal tissue. ILC2s stimulated by IL-33 and retinoic acid are transdifferentiated to ILCregs, producing IL-10 to suppress ILC2s and CD4^+^ T cell proliferation (84). In addition, Nakamura et al. demonstrated that fibroblasts acquire retinoic acid-producing capacity in transitioning to myofibroblasts in several kidney injury models (85). These findings indicate that ILC2-to-ILCreg plasticity is common during kidney injury, leading to the progression of renal fibrosis. These are therefore potential therapeutic targets as TGF-β source cells, although the full contribution of ILCregs to renal fibrosis is still enigmatic.

## Concluding and Future Perspective

The relationship of IL-33 and ILC2s has pivotal roles in renal immune homeostasis and kidney diseases leading to renal fibrosis. Although the functions of ILC2 in kidney diseases are gradually understood, there are still obscure in humans. However, it is intriguing that change of circulating ILCs correlate with the severity of some renal diseases. Moreover, it is interesting question what the difference means that ILC2 is dominant in murine kidney but ILC3 is dominant in human kidney, and it would be necessary to further investigate in detail. When these questions are clarified, it may be possible to elucidate the role and characteristics of ILC2 in the kidney and apply it to new therapeutic targets and clinical diagnosis in renal diseases.

## Author Contributions

RN and MI wrote the manuscript. All authors contributed to the article and approved the submitted version.

## Conflict of Interest

The authors declare that the research was conducted in the absence of any commercial or financial relationships that could be construed as a potential conflict of interest.

## Publisher’s Note

All claims expressed in this article are solely those of the authors and do not necessarily represent those of their affiliated organizations, or those of the publisher, the editors and the reviewers. Any product that may be evaluated in this article, or claim that may be made by its manufacturer, is not guaranteed or endorsed by the publisher.

## Supplementary Material

The Supplementary Material for this article can be found online at: https://www.frontiersin.org/articles/10.3389/fimmu.2021.688647/full#supplementary-material
