# Supplementary material for: The Roles of Kidney-Resident ILC2 in Renal Inflammation and Fibrosis
Source: Front Immunol. 2021 Jul 26;12:688647. doi: 10.3389/fimmu.2021.688647 (PMC8350317; doi:10.3389/fimmu.2021.688647)
Supplement: Supplementary file 1 [file Table_1.docx]

Table1. Murine ILC2 in kidney

Model	Characteristics	Outcome	Reference
UUO	CD45+ Lin- Thy1.2+ GATA3+	UUO induce infiltration and expansion of ST2+ immune cells including ILC2s<br>Upregulation of IL-33 following obstructive renal injury induces type 2 immune responses and UPJ urothelium hyperplasia	25
IRI	CD45+ Lin- IL-7Ra+ CD90.2+ ST2+	The severity of experimental renal IRI was irrespective of ILC2	27
IRI	CD45+ Lin- CD127+ ST2+ GATA3+	Post- or Pre-IL-33 treatment protected against IRI, and induced ILC2s, M2 macrophages, and Tregs<br>Depletion of ILC2 or M2 macrophage was disable IL-33-mediated renoprotection<br>ILC2 protected against IRI in an Areg-dependent manner	29
IRI	Lin- Thy1.2+ ST2+	IL-233 treatment increased ILC2 in blood and kidney, and protected from IRI<br>Adaptive transfer of ex-vivo cultured with IL-233 rescured from IRI injury	33
IRI	CD45+ Lin- ST2+ IL-7Ra+ (ILC2)<br>CD45+ Lin- ST2- IL-7Ra- c-kit+ (MPP type2)	IL-25 elicits ILC2 and MPPtype2 in kidny, and adoptive transfer of these cells attenuate IRI	32
AN	CD45+ Lin- CD127+ CD09.2+ GATA3	Exogeneous IL-33 induce ILC2 expansion in kidney and ameriorate kidney injury caused by AN<br>Eosinophils are required for IL-33–mediated renal tissue protection	28
AN	Lin- Thy1.2+ ST2+	Pre-treatment of IL-233 protected from doxorubicin-induced nephrotoxic injury	31, 33
DN	Lin- Thy1+ ST2+	The protection correlated with greater accumulation of Tregs, ILC2, alternately activated macrophages and eosinophils in the adipose tissue, along with a skewing toward Th2responses	30
